# Bupropion-Induced Delayed Hypersensitivity Serum Sickness-Like Reaction

**DOI:** 10.7759/cureus.36158

**Published:** 2023-03-14

**Authors:** Alyssa M Cartwright, Justin T Shaw, Dean Traiger

**Affiliations:** 1 Internal Medicine, Florida International University, Herbert Wertheim College of Medicine, Miami, USA; 2 Family Medicine, Physicians' Primary Care of Southwest Florida, Cape Coral, USA

**Keywords:** adverse drug reaction, serum sickness-like, delayed hypersensitivity, antidepressant, bupropion

## Abstract

Bupropion is an antidepressant utilized widely for the treatment of various mood disorders and smoking cessation due to its favorable side effect profile, cost, and response to therapy. While serious adverse reactions are rare, in the decades since its approval by the FDA, multiple cases of serum sickness-like reactions to bupropion have been reported, amongst other adverse drug reactions (ADRs). This report is of the case of a 25-year-old female who developed a serum sickness-like reaction to bupropion 21 days after initiation of treatment. She did not respond to conservative therapy but did respond promptly to oral corticosteroids and discontinuation of bupropion. This case serves to bolster the existing literature surrounding ADRs to bupropion and other antidepressant medications in the categories of systemic and dermatologic manifestations.

## Introduction

This case report describes the clinical presentation of a serum sickness-like reaction associated with a frequently prescribed atypical antidepressant medication (bupropion hydrochloride (HCl)), its diagnosis, and subsequent management. Bupropion is a norepinephrine/dopamine reuptake inhibitor that is commonly used to treat various mood disorders, most notably major depressive disorder and seasonal affective disorder. It can also be prescribed for smoking cessation, attention-deficit/hyperactivity disorder (ADHD), and obesity [1]. While this drug is generally well-tolerated, patients can occasionally present with an adverse drug reaction (ADR) similar to serum sickness. It is anticipated that raising awareness of this ADR will assist clinicians in recognizing and managing patients presenting similarly in the setting of a recently initiated potential offending medication.

## Case presentation

**Figure 1:**
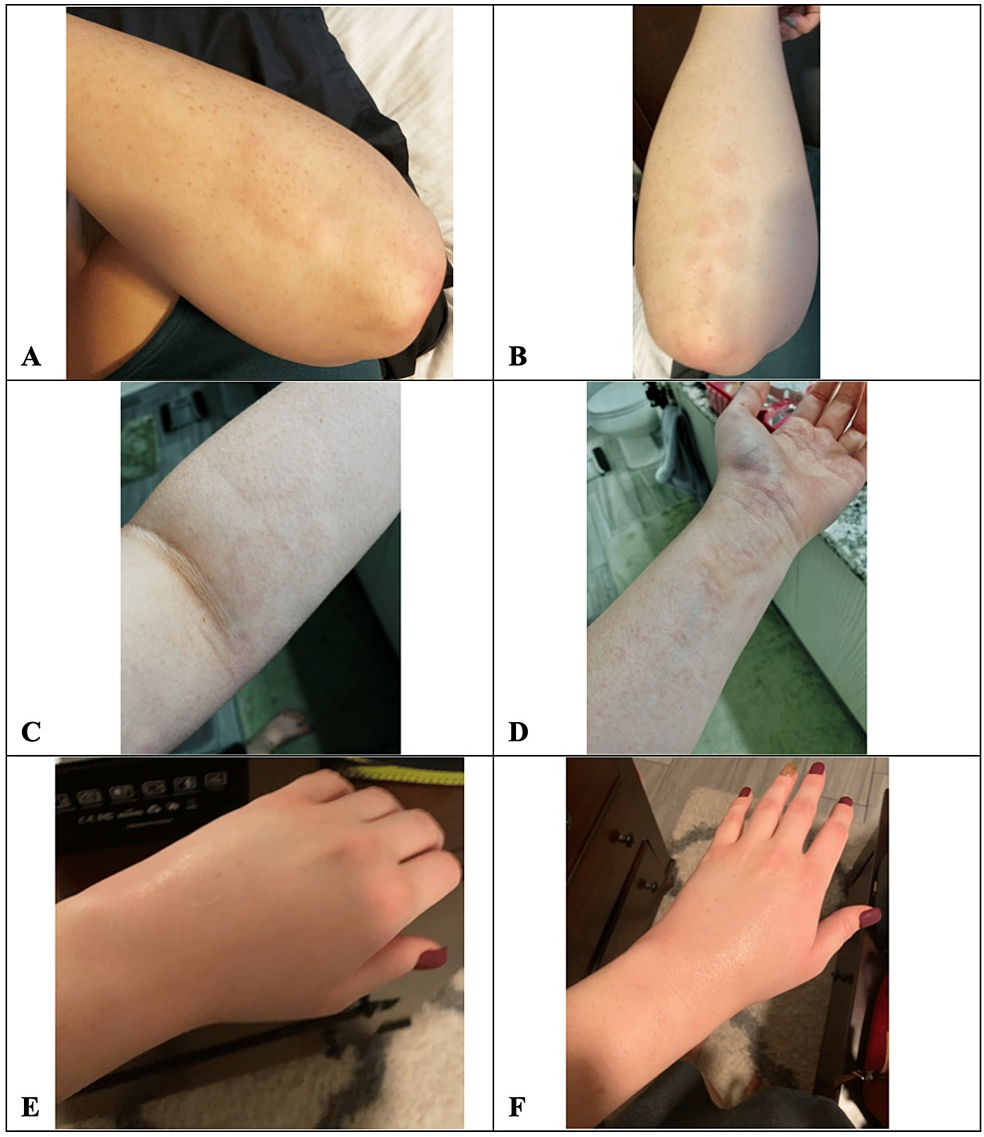
Clinical manifestations of delayed hypersensitivity to bupropion (A-B): Non-pruritic, diffuse maculopapular lesions with which patient awoke; (C-D): Urticarial wheals began to develop and migrate as the day progressed; (E-F): Diffuse angioedema of the bilateral upper extremities that developed several hours after the onset of urticarial wheals

On September 9, 2021, a 25-year-old female with a medical history of generalized anxiety disorder and major depressive disorder presented to her primary care physician (PCP) for evaluation of the two-day history of rash, angioedema, and polyarthralgias. The patient reported she woke up in the early morning of September 8, 2021, with symmetrically grouped papules on her upper extremity distal extensor surfaces without any associated pruritis or pain (Figures 1A, 1B). Hours later, in the mid-afternoon, she reported she became diffusely pruritic over her entire body, which was not alleviated with a cool shower or topical hydrocortisone cream. By the evening, she reported urticarial wheal formation with increasing pruritis, symmetric polyarthralgias of the bilateral metacarpophalangeal (MCP) joints, wrists, elbows, and shoulders, and significant angioedema of her bilateral upper extremities (Figures 1C-1F). Her symptoms persisted despite over-the-counter diphenhydramine 25 mg, ibuprofen 200 mg, and the application of topical hydrocortisone cream every four hours. The next morning, the patient woke up to discover that similar lesions had appeared on her face, torso, trunk, and flexor surfaces, and began to coalesce along with persistent intense pruritis. She denied fever, chills, nausea, vomiting, diarrhea, difficulty breathing at rest or on exertion, chest pain, sore throat, dysphagia, odynophagia, changes in vision, muscle weakness, recent illness, recent antibiotic use, recent changes in soaps or detergents, use of new perfumes or lotions, recent travel, recent outdoor activity, recent dietary changes, history of any other known allergies, or history of skin conditions.

## Discussion

Due to her worsening condition and failure of conservative treatment, the patient presented to her PCP for evaluation on September 9, 2021. After conducting a thorough history and physical, it was found that the patient was started on bupropion HCl 150 mg once daily 21 days before the onset of symptoms. She had never experienced similar symptoms, and the only recent lifestyle change she implemented was the addition of this antidepressant medication. As such, the patient was diagnosed clinically as experiencing an adverse reaction to the bupropion HCl. No laboratory studies or imaging tests were conducted due to the clinically diagnostic nature of drug-induced serum sickness-like reaction. For further evidence supportive of a drug-induced reaction, the Naranjo algorithm (advanced drug reaction probability scale) was employed, resulting in a score of six. This score indicated the patient’s clinical presentation was probably the result of a drug reaction (Table 1) [2]. She was prescribed a course of oral prednisone 20 mg for three days and counseled to stop taking the bupropion HCl. Three hours after taking the first dose of prednisone, the patient reported significant improvement in pruritis, angioedema, urticaria, and arthralgias with full resolution of symptoms reported within one day of oral steroid initiation and cessation of the suspected offending bupropion HCl. The patient in the months following this presentation has not restarted bupropion HCl and has not complained of similar symptoms since during routine follow-up.

**Table 1:** Naranjo scale for estimation of probability of adverse drug reaction Score ≤ 0 = unlikely that an adverse drug reaction has occurred; Score 1-4 = likely that an adverse drug reaction has occurred; Score 5-8 = probably an adverse drug reaction has occurred; Score ≥ 9 = definitively an adverse drug reaction has occurred

Questionnaire	Yes	No	Unknown/Not Done	Score
1. Are there previous conclusive reports on this reaction?	+1	0	0	+1
2. Did the adverse event appear after the suspected drug was administered?	+2	-1	0	+2
3. Did the adverse event improve when the drug was discontinued, or a specific antagonist was administered?	+1	0	0	+1
4. Did the adverse reaction reappear when the drug was readministered?	+2	-1	0	0
5. Are there alternative causes (other than the drug) that could on their own have caused this reaction?	-1	+2	0	+2
6. Did the reaction reappear when a placebo was given?	-1	+1	0	0
7. Was the drug detected in the blood (or other fluids) in concentrations known to be toxic?	+1	0	0	0
8. Was the reaction more severe when the dose was increased or less severe when the dose was decreased?	+1	0	0	0
9. Did the patient have a similar reaction to the same or similar drugs in any previous exposure?	+1	0	0	0
10. Was the adverse event confirmed by any objective evidence?	+1	0	0	0
Total score				6

Current literature

Delayed hypersensitivity in the setting of bupropion administration is not novel. Initially approved in 1985, bupropion HCl prescribing information included known reports of serum sickness-like symptoms that were suggestive of delayed hypersensitivity [3]. Drug-induced rashes and other systemic reactions to psychotropic medications vary widely in severity, ranging from mild to life-threatening [4]. It has been described that the incidence of bupropion-induced dermatologic reactions has a prevalence of 1-4% [5]. However, the incidence of serum sickness-like reactions may be higher than some clinicians believed in regular practice as of a few decades ago [6].

Multiple case reports have demonstrated the wide severity of such reactions, with some patients experiencing remission of symptoms in the outpatient setting and others requiring hospitalization amid the insidious onset of symptoms similar to the case presented here [6-9]. Additionally, a Taiwanese nationwide cohort study found the risk of urticaria to be higher in bupropion users than in matched controls within four weeks of starting the medication; the average onset of urticaria occurred 15-28 days after initiating bupropion [10].

Recommendations for the treatment of patients experiencing a rare serum sickness-like reaction to bupropion have included the use of antihistamines, non-steroidal anti-inflammatory drugs (NSAIDs), topical corticosteroids, systemic corticosteroids if needed, and prompt discontinuation of bupropion. Prior case series have suggested that such reactions typically resolve within around 10 days of treatment [11].

Cases of bupropion-induced serum sickness-like reactions have been reported previously. Serious adverse reactions (SARs) to bupropion were characterized in a French analysis of reported reactions since marketing authorization for bupropion in 2001. This analysis of reported adverse reactions over a three-year timeframe included 40 cases of serum sickness-like reactions out of an exposed population of 692,798 (< 0.006%) [12].

Previously reported cases of bupropion-induced serum sickness-like reactions have demonstrated strikingly similar clinical presentations as described in this case. Notably, existing case reports have described marked improvement in systemic symptoms (angioedema, pruritis) within hours of initiation of treatment (corticosteroids, antihistamine, cessation of suspected offending drug) [13-14]. While angioedema is not a common adverse reaction to bupropion, few cases have been reported and this case report serves to bolster the existing literature regarding this specific adverse drug reaction.

## Conclusions

While bupropion is generally a well-tolerated medication for various mood disorders, it has been known to cause delayed hypersensitivity serum sickness-like reactions in a small percentage of patients. Symptoms of this reaction can include pruritis, urticaria, angioedema, and polyarthralgias, which can be very concerning for patients given the sudden onset of symptoms and the potential for rapid evolution and failure of symptoms to resolve spontaneously. Discontinuation of the offending agent with the use of conservative measures such as oral antihistamines and topical steroid creams have been shown to aid in the resolution of such symptoms. However, refractory or severe cases can be managed with systemic corticosteroids. Clinicians may consider counseling patients regarding the possibility of this adverse reaction when initiating bupropion with prompt discontinuation and consideration of initiation of various other therapies for their underlying conditions if such a reaction occurs.
